# “*Out of the Can*”: A Draft Genome Assembly, Liver Transcriptome, and Nutrigenomics of the European Sardine, *Sardina pilchardus*

**DOI:** 10.3390/genes9100485

**Published:** 2018-10-09

**Authors:** André M. Machado, Ole K. Tørresen, Naoki Kabeya, Alvarina Couto, Bent Petersen, Mónica Felício, Paula F. Campos, Elza Fonseca, Narcisa Bandarra, Mónica Lopes-Marques, Renato Ferraz, Raquel Ruivo, Miguel M. Fonseca, Sissel Jentoft, Óscar Monroig, Rute R. da Fonseca, L. Filipe C. Castro

**Affiliations:** 1 Interdisciplinary Centre of Marine and Environmental Research (CIIMAR), University of Porto, 4450-208 Matosinhos, Portugal; andre.machado@ciimar.up.pt (A.M.M.); alvarinacouto@gmail.com (A.C.); campos.f.paula@gmail.com (P.F.C.); fonseca.ess@gmail.com (E.F.); monicaslm@hotmail.com (M.L.-M.); renatobarbosaferraz@gmail.com (R.F.); ruivo.raquel@gmail.com (R.R.); mig.m.fonseca@gmail.com (M.M.F.); 2 Centre for Ecological and Evolutionary Synthesis (CEES), Department of Biosciences, University of Oslo, 0371 Oslo, Norway; o.k.torresen@ibv.uio.no; 3 Department of Aquatic Bioscience, The University of Tokyo, Tokyo 113-8654, Japan; naokikby@g.ecc.u-tokyo.ac.jp; 4 The Bioinformatics Centre, Department of Biology, University of Copenhagen, DK-2200 Copenhagen, Denmark; 5 Natural History Museum of Denmark, University of Copenhagen, DK-1350 Copenhagen, Denmark; bent.petersen@snm.ku.dk; 6 Centre of Excellence for Omics-Driven Computational Biodiscovery, Faculty of Applied Sciences, Asian Institute of Medicine, Science and Technology, Kedah 08000, Malaysia; 7 Portuguese Institute for the Sea and Atmosphere, (IPMA), 1749-077 Lisbon, Portugal; monicafelicio124@hotmail.com (M.F.); narcisa@ipma.pt (N.B.); 8 Department of Biology, Faculty of Sciences, University of Porto, 4099-022 Porto, Portugal; 9 Institute of Biomedical Sciences Abel Salazar (ICBAS), University of Porto, 4099-022 Porto, Portugal; 10 Centre for Coastal Research, Department of Natural Sciences, University of Agder, 4630 Kristiansand, Norway; 11 Instituto de Acuicultura Torre de la Sal, Consejo Superior de Investigaciones Científicas (IATS-CSIC), 12595 Ribera de Cabanes, Spain; 12 Center for Macroecology, Evolution, and Climate, Natural History Museum of Denmark, University of Copenhagen, DK-2100 Copenhagen, Denmark

**Keywords:** European sardine, draft genome, teleosts, comparative genomics, long chain polyunsaturated fatty acids

## Abstract

Clupeiformes, such as sardines and herrings, represent an important share of worldwide fisheries. Among those, the European sardine (*Sardina pilchardus*, Walbaum 1792) exhibits significant commercial relevance. While the last decade showed a steady and sharp decline in capture levels, recent advances in culture husbandry represent promising research avenues. Yet, the complete absence of genomic resources from sardine imposes a severe bottleneck to understand its physiological and ecological requirements. We generated 69 Gbp of paired-end reads using Illumina HiSeq X Ten and assembled a draft genome assembly with an N50 scaffold length of 25,579 bp and BUSCO completeness of 82.1% (Actinopterygii). The estimated size of the genome ranges between 655 and 850 Mb. Additionally, we generated a relatively high-level liver transcriptome. To deliver a proof of principle of the value of this dataset, we established the presence and function of enzymes (Elovl2, Elovl5, and Fads2) that have pivotal roles in the biosynthesis of long chain polyunsaturated fatty acids, essential nutrients particularly abundant in oily fish such as sardines. Our study provides the first omics dataset from a valuable economic marine teleost species, the European sardine, representing an essential resource for their effective conservation, management, and sustainable exploitation.

## 1. Introduction

**Figure 1:**
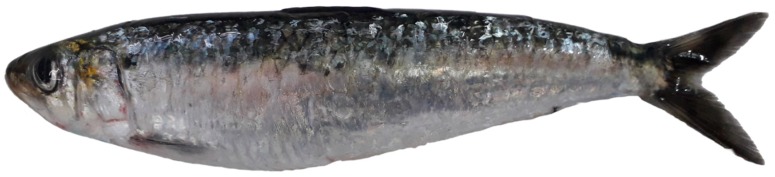
Photograph of a specimen of European sardine, *Sardina pilchardus* (photograph credits to Mónica Felício and André M. Machado).

Teleosts comprise the most species-rich group of vertebrates, with approximately 30,000 described species [1]. During the last decades, teleosts emerged as particularly insightful models for comparative evolutionary studies [2]. Moreover, numerous teleost fish species are of high commercial importance for both fisheries and aquaculture. Fish are not only an important source of protein for human consumers, but oily species represent unique sources of the healthy omega-3 long-chain polyunsaturated fatty acids (LC-PUFAs), which have been shown to have essential roles in cardiovascular health and neuronal development [3,4]. Yet, over-fishing combined with global changes entail countless threats to this taxon, making it an interesting target for aquaculture which already provides approximately half of the seafood consumed worldwide [5]. The oily fish European sardine (*Sardina pilchardus*, Walbaum 1792) (Figure 1) is one of the most commercially important species [6], particularly for the canning industry, and has high nutritional value primarily linked to its omega-3 LC-PUFA content. Interestingly, a steady and sharp decline in capture levels has been observed in the last decade, which is currently dictating severe cuts in fishing quotas within the European Union [7]. Recent advances in captive sardine culture practices represent promising research possibilities [8,9]; however, the complete absence of genomic resources from this iconic species imposes a severe bottleneck. Genome data emerging from high-throughput sequencing technology combined with multiple assembly algorithms has represented a truly transformative event in the field of comparative genomics [10]. Moreover, de novo assemblies based on low coverage and short read approaches are cost effective, and provide valuable biological information [11,12,13]. Here, we present the first draft genome of the European sardine and provide a relatively high-level liver transcriptome enabling nutrigenomics studies in this iconic species. The genomic makeup of the European sardine was further compared to that of other teleosts, including the closely related clupeid, the Atlantic herring [14]. As a proof of principle, we selected the LC-PUFA biosynthesis, a metabolic pathway accounting for the production of omega-3 fatty acid in fish [15], and characterized key genes with well-established roles within these pathways [3,16].

## 2. Methods, Results and Discussion

### 2.1. Sampling, DNA Extraction, Library Preparation and Genome Sequencing

One *S. pilchardus* specimen (Figure 1) was caught off Esposende (41.501944 N 8.851667 W), Portugal, under the “Programa Nacional de Amostragem Biológica” carried out by the Instituto Português do Mar e da Atmosfera (IPMA). Tissues were harvested immediately and stored in 100% ethanol (muscle) and RNA later (liver) until further processing (Supplementary Table S1 in Supplementary File 2). Genomic DNA was extracted from muscle tissue (~0.5 g) in three replicates, using Qiagen’s DNeasy Blood & Tissue Kit (Valencia, CA, USA) according to the manufacturer’s instructions, with the following modifications: prior to elution in 100 μL AE buffer, samples were incubated at 37 °C for 10 min, to increase DNA yield. DNA concentration and integrity were verified using an Agilent Genomic DNA ScreenTape (Waldbronn, Germany). We constructed one 150 bp paired-end reads library from 1.2 μg of genomic DNA using the standard Illumina protocol for the TruSeq Nano DNA library kit (Illumina, San Diego, CA, USA). Sequencing was performed with the Illumina HiSeq X Ten (Macrogen, Seoul, Korea) platform and generated 69.0 Gbp of raw reads for downstream analysis (Supplementary Table S1 in Supplementary File 2).

### 2.2. RNA Extraction, Library Preparation and Sequencing

Total RNA was extracted from liver using Illustra RNAspin Mini RNA Isolation Kit (GE Healthcare, Hammersmith, UK) according to the manufacturer’s instructions. The isolated RNA was treated with RNase-free DNase I and eluted with RNase-free water. A strand-specific library with insert size of 250~300 bp was built after conversion of the liver total RNA to cDNA, and sequenced using 150 bp paired-end reads on the Illumina HiSeq 2500 platform by Novogene (Beijing, China). A total of 122.8 million raw reads were produced (Supplementary Table S1 in Supplementary File 2).

### 2.3. RNA-Seq Raw Data Clean-Up and De Novo Assembly Transcriptome

The quality of raw RNA-Seq reads were scrutinized using the FastQC software (http://www.bioinformatics.babraham.ac.uk/projects/fastqc/). Trimmomatic (v0.36) [17] was used to clean up the initial dataset (LEADING:15 TRAILING:15 SLIDINGWINDOW:4:20 MINLEN:50) (Table 1). To assemble the paired end reads we used the de novo assembler Trinity (v2.4.0) [18]. All default parameters were used except for SS_lib_type—RF and min_contig_length of 300 bp. To check for contamination sources such as vectors, adapters or other exogenous sequences, we applied the same methodology as previously described [19]. Thus, using the MCSC (Model-based Categorical Sequence Clustering) decontamination pipeline [20] and UniVec database (build 10.0), we obtained the final decontaminated transcriptome assembly further used in the genome annotation process. Overall, from 111.5 million clean reads we obtained a total of 245,053 assembled transcripts, with N50 of 1760 bp (Table 1 and Supplementary Table S1 in Supplementary File 2). Additionally, we also evaluated the gene content completeness using the Benchmarking Universal Single-Copy Orthologs (BUSCO (v.3)) [21]. This analysis was done against the lineage-specific library of *Actinopterygii*, and showed that from 4584 BUSCO ortholog genes, our transcriptome assembly contains 80.6% of the sequences (complete and partial; Table 1).

### 2.4. DNA Raw Data Clean-Up and Genome Size Estimation

Raw Illumina reads were first processed with Trimmomatic [17] for the removal of adapter sequences, and trimming bases with quality <20 and discarded reads with length <80. The genome size estimation was performed with two different approaches, using GenomeScope (v1.0.0) [22] and Kmergenie (v1.7044) [23] on genomic clean reads. The first approach requires the Jellyfish (v2.2.6) software to build k-mer frequency distributions. We applied three values of k-mers, 21, 25, and 31, and each histogram was submitted to the GenomeScope software. In the end, we estimated a genome size between 625–637 Mbp, heterozygosity levels between 1.60–1.75%, and unique content of 85.0–85.7% (Supplementary Figure S1A–C in Supplementary File 1 and Supplementary Table S2 in Supplementary File 2). The Kmergenie software with the diploid model was also used, and a genome size of 850 Mb was estimated.

### 2.5. Assembly and Assessment of Sardine Genome

The genomic clean reads were assembled with the Celera assembler (downloaded from the CVS Concurrent Version System, http://wgs-assembler.sourceforge.net/) repository on 21 June 2017; for details see Supplementary Materials and Methods in Supplementary File 1. Interestingly, this assembler has been successful for other teleost species genomes (e.g., *Parachaenichthys charcoti*) [24]. After genome assembly, the clean reads were back-mapped to the sardine genome with BWA mem [25]. PCR duplicates were removed with Picard MarkDuplicates (http://picard.sourceforge.net) and local realignment around indels was done with GATK [26]. A median insert size of 441 bp was determined with Picard CollectInsertSizeMetric. To evaluate the genome assembly, we primarily used QUAST v4.3 [27]. Next, the validation of the genome was done with the K-mer analysis toolkit (KAT) [28]. Through this analysis, it was possible to check how the Celera Assembler dealt with the heterozygosity of the sardine. In Supplementary Figure S2 of Supplementary File 1, two peaks can be observed: the first at 25× (heterozygotic) and the second at 55× (homozygotic). Ideally, it is expected that, after the assembly, the shared k-mers contents of both distributions (red zones) are merged (black zones in the first peak), and stayed represented just once in both distributions [28]. Notwithstanding, our distributions show this profile, with the black content of the first peak nearly filling the full area of the second peak. Second, and similarly to the transcriptomic approach, the sardine genome was also inspected in terms of expected gene content with the BUSCO v3 software [21]. From the total of 4584 genes present in the Actinopterygii library, we found 82.1% (complete and fragmented). Importantly, the BUSCO scores are in line with previous reports for other fish assemblies with similar methodologies and sequencing strategies. For instance, Malmstrøm and colleagues [13] reported the genome of 66 teleost species, in which 48 genomes showed between 33 and 84% (complete and fragmented) of the genes present in the Actinopterygii library. A combination of short and long read provides, however, a higher detection of BUSCO scores, and should be pursued in the near future [29,30,31,32]. To further determine the genome completeness, we mapped the de novo assembled liver transcriptome against the genome with Blat [33]. More than 97% of the transcripts have a match hit with at least one genomic scaffold, and 89% of the total number of bases are covered by our assembly (Supplementary Table S3 in Supplementary File 2).

### 2.6. Genome Annotation

The genome annotation of sardine was performed using two-pass iterative MAKER (v2.31.9) pipeline [34]. Prior to running Maker, we identified repetitive sequences in our genome assembly using an approach described in [35]. Briefly, RepeatModeler (v1.0.8) [36], LTRharvest [37] part of Genome tools (v1.5.7) [38] and TransposonPSI [39] were used in combination to create a set of putative repeats. Elements with a single match against a UniProtKB/SwissProt database and not against the database of known repeated elements included in RepeatMasker were removed. The remaining elements were classified and combined with known repeat elements from RepBase (release 20150807) [40]. Then, the custom repeat database RepBase-derived and RepeatMasker library (release 20150807) [40] were used in the RepeatMasker (v4.0.6) [41] inside the Maker pipeline. In addition to the previously-described Trinity-based transcriptome assembly, the transcriptome reads were mapped to the genome with HISAT (v2.0.5) [42,43] and assembled with StringTie (v1.3.1) [44]. The mapped reads were used to train the GeneMark-ET (v4.32) and AUGUSTUS (v3.2.3) [45] ab initio gene predictors via the tool BRAKER (v1.11) [46]. Splice junctions were detected from the mapped reads with Portcullis (v1.02) [47], and these were used as input to Mikado (v1.2.2) [48], together with the StringTie and Trinity transcriptome assemblies, to merge the redundant transcripts. The resulting GFF file was used as input to Maker (as est_gff). The predicted genes from GeneMark-ET were also used as input to Maker (as pred_gff), but only in the first iteration, since Maker keeps the gene names as given by GeneMark-ET, and uses them in the output GFF of Maker, which can cause issues with downstream analysis. The first iteration of Maker (which also included proteins from the UniProtKB/SwissProt, cleaned of transposable element proteins) was used to train SNAP (v2013_11_29) [49] with the transcriptome, protein evidence, and custom library of repeats. In the second iteration, we did not use the GeneMark-ET predictions, but utilized AUGUSTUS and SNAP, in addition to the UniProtKB/SwissProt database and the transcriptome.

To functionally annotate the genes and protein models, we used two independent approaches. First, we used a BLAST (v2.6.0) [50] methodology with the following parameters (blast-p, -evalue 1e−5, -seg yes, -soft masking true, -lcase masking, and -num_alignments 1), against the UniProtKB/SwissProt database. In the second approach, we opted for the inclusion of InterProScan [51] searches. The outputs of both approaches were used to refine the gene and protein models, as established in protocols of Campbell et al. (2014) [34]. Finally, our dataset contained 29,701 genes, which were selected based on a maximum Annotation Edit Distance (AED) score of ≤0.5 (from 0 to 1, where the 0 corresponds to strong evidence support, and 1 corresponds to no evidence support) (Table 1). The number of predicted coding genes in our dataset is higher than that previously calculated for the *Clupea harengus* genome (23,336 coding gene models) [14]. This discrepancy likely reflects a higher level of gene fragmentation in our assembly, which does not impact the application of the dataset for experimental research (see Section 2.9).

**Figure 2:**
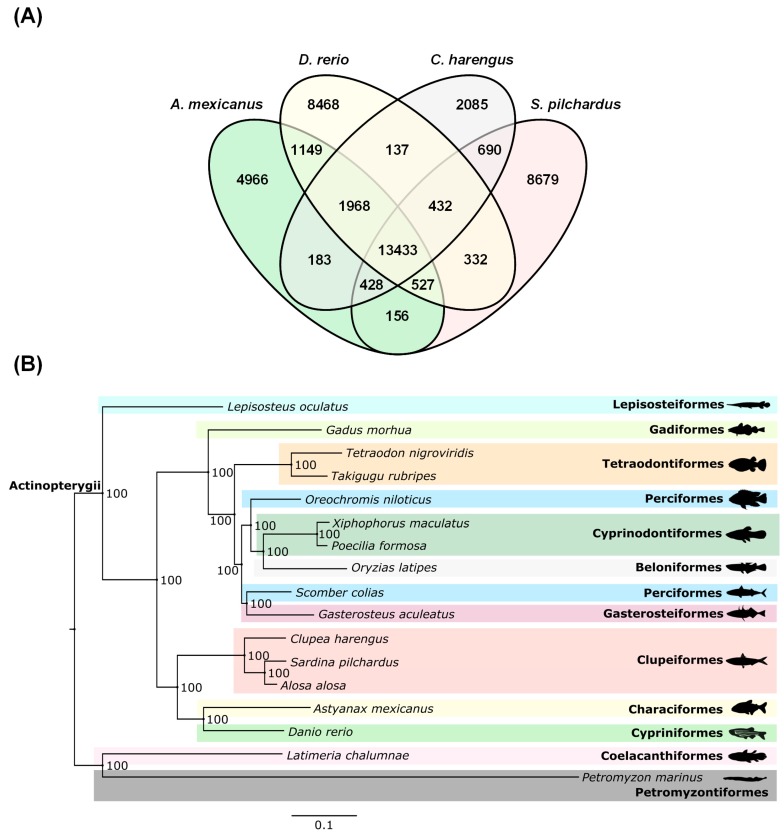
Genome evolution and phylogenomics. (**A**) Orthologous gene families across four fish genomes (European sardine, zebrafish, herring and blind cave fish). (**B**) Phylogeny of vertebrates (lamprey as the outgroup species); numbers at nodes represent bootstrap values.

To obtain a broad overview of the annotated gene repertoire in the Clupeidae family, we also compared the ortholog gene collection of sardine with other teleost species including another clupeid, *C. harengus* [14], and to two well-annotated genomes from the sister clade, the Otophysa [2] (for details see Supplementary Materials and Methods in Supplementary File 1). Using the Orthofinder v2.2.6 [52], we identified 24,677 clusters of orthologs genes in the sardine genome: 13,433 orthogroups were shared among the four species, and at least 690 orthogroups were shared exclusively between sardine and herring of the Clupeidae family (Figure 2A). A total of 8679 orthogroups were found to be exclusive to sardine, with this number likely reflecting gene fragmentation in the assembly process, a consequence of the sequencing strategy.

### 2.7. Sardine Phylogenomics

The phylogenomic analysis was conducted with gene orthologues from 17 fish species, representing 13 orders, providing an ample representation of teleost diversity, with a specific focus in Clupeidae. Transcriptomic sequences for *S. pilchardus*, *C. harengus*, *Alosa alosa*, and *Scomber colias* were clustered based on sequence similarity with zebrafish sequences. Briefly, a blast-p output was filtered to obtain only hits with a percent identity equal or higher than 50% and a length of at least 30 amino acids. We then selected the hit with the highest bitscore. Zebrafish orthologs were retrieved from the ENSEMBL database [53] for all the 17 fish species available, and assigned to the respective cluster. In order to avoid paralogs, only clusters with one sequence per species were considered, resulting in 106 ortholog clusters that included all species (Supplementary File 3). Amino acid sequences were then aligned with MAFFT v7.402 [54] using the model L-INS-i, recommended for a small number of sequences with long gaps. The resulting 106 sequences alignments were then concatenated (42,267 bp long). A maximum-likelihood phylogenetic inference for the concatenated protein alignment was done in ExaML v3 [55], including 100 bootstrap replicates, under the protgammaauto option, and was computed using parsimony starting trees for each replicate, using RAxML v8.2.12 [56]. In the ExaML tree, two major groups can be observed: one that comprises all Actinopterygian, and another with the Sarcopterygii and Cephalaspidomorphi in the basal position of the tree (Figure 2B). In this tree, all species belonging to the Clupeiformes order are clustered together, and the same for Tetraodontiformes and Cyprinodontiformes. Perciformes are the only order that is separated into two, with *Oreochromis niloticus* closely related to Cyprinodontiformes and *S. colias* with Gasterosteiformes. The position of *Lepisosteus oculatus* at the base of the Actinopterygian cluster was also recovered with maximum support. Overall, our phylogenetic analysis demonstrates the phylogenetic position of the European sardine, together with other clupeids such as the allis shad (*A. alosa*) and the Atlantic herring (Figure 2B). The same general phylogenetic relationships were recovered when the concatenated mitochondrial dataset of protein-coding genes was used. The only exception position was the *A. mexicanus*, in that it was clustered together (with low statistical support) with Clupeiformes and not with zebrafish (Supplementary Materials and Methods and Supplementary Figure S4 of Supplementary File 1, and Supplementary Table S4 in Supplementary File 2).

### 2.8. Mitochondrial Genome

We used NOVOPlasty (v2.6.5) [57] to perform the de novo assembly of the sardine mitochondrial genome (mtDNA). The assembly was executed using the raw whole genome sequencing reads only with the adapters removed (authors’ instructions), and using a *cox1* mtDNA gene nucleotide sequence of the same species (NCBI accession number NC_009592.1 (5484 … 7034)). The k-mers length was set to 39, 50, and 75 bp, and all assembly runs resulted in the same mtDNA circular contig with total length of 17,755 bp. We also used NOVOPlasty to detect heteroplasmy in the newly-assembled mtDNA, with a minimum minor allele frequency option set to 0.01 (heteroplasmy detection of >1%). Two heteroplasmic positions were detected in the kmer-75 at positions 3500 (from T to G, alternative allele frequency of 1.23%, depth of coverage of 326, located in *mt-nd1* gene), and another at position 10208 (from T to C, alternative allele frequency of 1.02%, depth of coverage of 391, located in *mt-nad4l* gene). Mitochondrial gene annotations were performed using MITOS (v2) [58] and tRNAs gene limits were rechecked with ARWEN (v1.2) [59]. All typical Metazoan genes were annotated (13 protein coding genes, 22 transfer RNAs, and 2 ribosomal RNAs, Supplementary Figure S3 in Supplementary File 1). The complete mtDNA was deposited in GenBank (Supplementary Table S1 in Supplementary File 2).

### 2.9. Gene Orthologs of LC-PUFA Desaturation and Elongation Are Present in the Sardine Genome and Transcriptome

**Figure 3:**
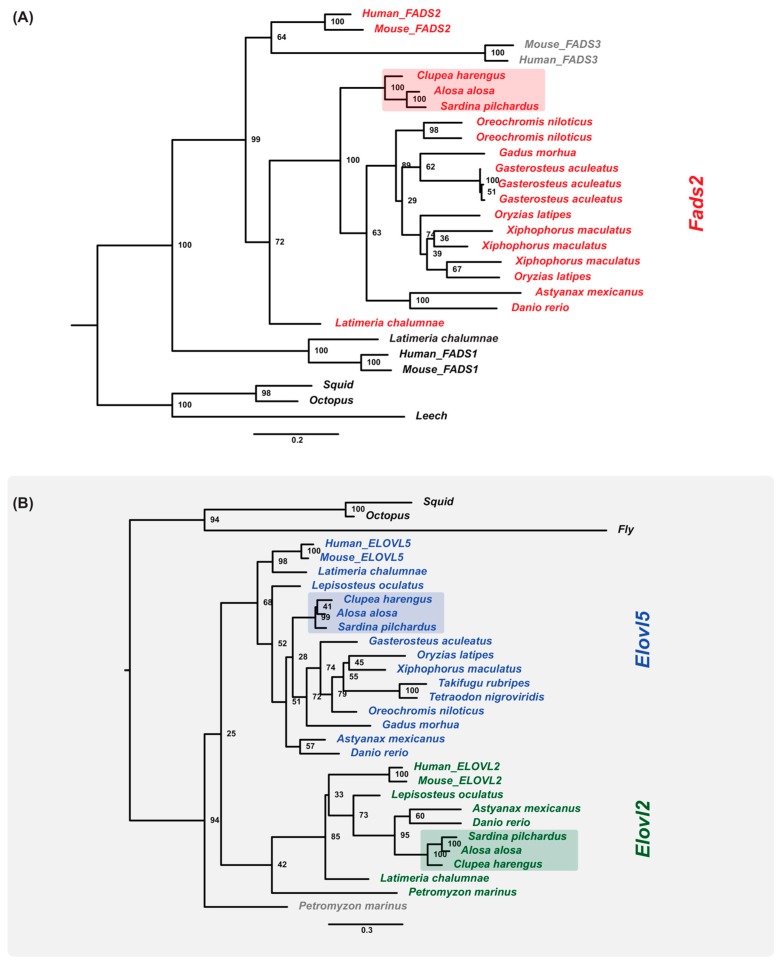

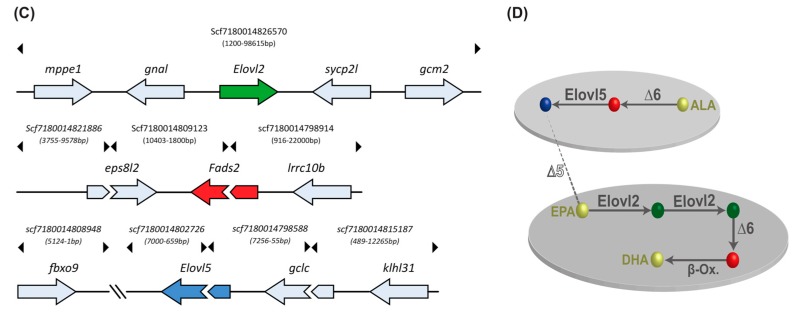
Maximum likelihood phylogenetic analysis of *fads2* (**A**) and *elovl* orthologues (**B**) analyzed in the present study: Clupeiformes species are highlighted, node numbers indicate bootstrap values. (**C**) Reconstructed genomic *loci* of *fads2*, *elovl2*, and *elovl5* denote synteny conservation between the European sardine and Atlantic herring: scaffold coordinates and identified neighbouring genes are indicated; broken lines and arrows denote reconstruction from overlapping scaffolds. (**D**) LC-PUFA biosynthesis pathway in the European sardine, dashed line indicates the Δ5 desaturation capacity absent in the European sardine, n-3 fatty acids are indicated in yellow: ALA—α-Linolenic acid (18:3n-3), EPA—eicosapentaenoic acid (20:5n-3) and DHA—docosahexaenoic acid (22:6n-3).

To demonstrate the biological value of the omics datasets, we next investigated the key enzymes of LC-PUFA biosynthesis in the sardine draft genome and liver transcriptome, a major metabolic site for PUFA metabolism [15]. More specifically, we investigated the repertoire and function of genes encoding fatty acyl desaturases (Fads) and elongation of very long chain fatty acid (Elovl) proteins with pivotal roles in LC-PUFA biosynthesis [3]. Among *fads*, our data shows that the European sardine possess one single *fads*-like gene that was confirmed to be orthologous of *fads2* (Figure 3A,C) (for details see Supplementary Materials and Methods in Supplementary File 1 and Supplementary File 4). Our microsynteny analyses shows the conservation of the reconstructed *locus*, when compared to *C. harengus*, and further suggests the absence of *fads1* from sardine’s genome, in agreement with the loss of this tandem gene duplicate during teleost evolution (Figure 3C, Supplementary Tables S5 and S6 in Supplementary File 2) [16]. Among *elovl*, we identified two *elovl*-like sequences, namely *elovl2* and *elovl5*, with well-known roles in LC-PUFA biosynthetic pathways (Figure 3B). Again, microsynteny conservation was found in the reconstructed *loci*. While *elovl5* is present in virtually all teleosts [3,60], *elovl2* has only been described in a few of species, and is reported to be lost in the Neoteleostei [60]. Thus, the presence of an *elovl2* gene in sardine is consistent with the phylogenetic location of this species within the Otomorpha group, to which species with characterized *elovl2* belong [61,62]. Accession numbers for the herein isolated *fads2*, *elovl2*, and *elovl5* gene orthologs have been deposited in GenBank (Supplementary Table S1 in Supplementary File 2), and are located within genome scaffold numbers: *fads2—*scaffolds scf7180014809123 and scf7180014798914; *elovl2*—scaffold scf7180014826570; and *elovl5*—scaffolds scf7180014798588 and scf7180014802726 (for details see Supplementary Materials and Methods in Supplementary File 1 and Supplementary Table S5 in Supplementary File 2).

We next examined the function of the enzymes encoded by the *fads2*, *elovl2,* and *elovl5* genes to establish their contribution to LC-PUFA biosynthesis in sardine using an established yeast-based expression system [63] (Figure 3D) (Supplementary Table S7 in Supplementary File 2; see Supplementary Materials and Methods in Supplementary File 1 for details). The sardine *fads2* encodes a desaturase with Δ6 and Δ8 desaturase activities (Supplementary Tables S8 and S9 in Supplementary File 2), typical of vertebrates Fads2 enzymes [3]. Both Elovl2 and Elovl5 were capable of elongating polyunsaturated fatty acids from 18 to 22 carbons, which is consistent with activities reported in other vertebrate orthologs (Supplementary Table S10 in Supplementary File 2). Such enzymatic capabilities enable sardines to produce docosahexaenoic acid (DHA) from eicosapentaenoic acid (EPA, 20:5n-3). However, the lack of Δ5 desaturation capacity strongly suggests that sardine is unable to produce EPA endogenously or arachidonic acid (ARA, 20:4n-6) (Figure 3D). Importantly, these results clearly illustrate the validity of the herein released omics datasets for nutrigenomic studies.

## 3. Conclusions

We generated a draft genome assembly and liver transcriptome of the commercially-important European sardine. We further demonstrate the power of this dataset by exploring the endogenous capacity of sardines (clupeids) to biosynthesize LC-PUFAs. The information retrieved here, and made publicly available, will further contribute not only to elucidating the fundamentals of the physiology, endocrinology, reproduction, and nutrition of sardine, providing an essential framework for future conservation and sustainable exploitation of this iconic species, but will also contribute to future comparative genomic studies, notably regarding life history strategies among teleosts. 

## Figures and Tables

**Table 1:** Summary of genome and liver transcriptome statistics of the European sardine, *Sardina pilchardus*.

Features	Genome #	Liver Transcriptome #
**Raw Data**		
Raw sequencing reads	456,775,568	122,806,922
Clean reads	412,914,751	111,524,231
**Contig statistics**		
Number of contigs	90,290	245,053
Total contig size, Mb	640.1	278.5
Contig N50 size, bp	10,878	1760
Longest contig, bp	87,474	15,773
GC/AT/N, %	44.45	48.10
**Scaffold statistics**		
Number of scaffolds	45,321	-
Total scaffold size, Mb	641.5	-
Scaffold N50 size, bp	25,577	-
Longest scaffold, bp	285,113	-
Genome coverage, ×	59	-
**BUSCO Completeness** **(Met/Ver/Actino)**		
Complete, %	82.7/70.5/68.8	99.1/80.6/72
Complete and single copy, %	78.8/68.4/66.3	41.5/31.2/29.1
Complete and duplicated, %	3.9/2.1/2.5	57.6/49.4/42.9
Fragmented, %	9.2/19.0/13.3	0.6/10.5/8.6
Missing, %	8.1/10.5/17.9	0.3/8.9/19.4
Total BUSCO found	91.9/89.5/82.1	99.7/91.1/80.6
**Annotation**		
Number of protein-coding genes	29,701	-
Number of functionally annotated proteins	28,783	-
Average CDS length	1561.42	-
Longest CDS	49,643	-
Average protein length	373.45	-
Longest protein	16,525	-
Average number of exon per gene	6.59	-

**#** All statistics are based on contigs/scaffolds of size ≥200 bp. Met: From a total of 978 genes of Metazoa library profile; Ver: From a total of 2586 genes of Vertebrata library profile; Actino: From a total of 4584 genes of Actinopterygii library profile.

## Supplementary Materials

The following are available online at http://www.mdpi.com/2073-4425/9/10/485/s1, Supplementary File 1 containing Supplementary Figures, Figure S1: Estimation of genome size, repeat content, and heterozygosity by GenomeScope, Figure S2: Assessment of the genome assembly by comparison of read spectrum and assembly copy number, Figure S3: Circular gene map of the mitochondrial genome of *Sardina pilchardus*, Figure S4: Phylogenetic tree of ray-finned fishes estimated from 13 concatenated individual mtDNA protein-coding gene amino acid sequences, Supplementary Materials and Methods, and Supplementary References. Supplementary File 2 containing Supplementary Tables, Table S1: MixS descriptors and accession numbers of tissue samples, raw data and Assemblies of *Sardina pilchardus,* Table S2: GenomeScope profile statistics for *Sardina pilchardus* WGS reads estimated using k-mer 21, 25, and 31, Table S3: Completeness assessment of sardine genome using the liver transcriptome, Table S4: List of species and GenBank references used in the mtDNA phylogeny, Table S5: Blast-n output of *Clupea harengus* sequences against the sardine genome, Table S6: Top results from a reciprocal blast-p search of the 5 proteins flanking the genomic locations of *fads2*, *elovl2* and *elovl5* in herring and the sardine genomes, Table S7: Primer sequences and PCR conditions used to isolate *fads2*, *elovl2* and *elovl5* genes, Table S8: Functional characterization of *Sardina pilchardus Elovl2* and *Elovl5*, Table S9: Functional characterization of *Sardina pilchardus* Fads2, Table S10: *Sardina pichardus* Fads2 + Zebrafish Elovl2 co-expression. Supplementary File 3 containing Clusters of sequences used to Sardine Phylogenomics Analyses. Supplementary File 4 containing Clusters of sequences used to—Gene orthologs of LC-PUFA desaturation and elongation are present in the sardine genome and transcriptome—analyses. The raw sequencing data (RNA-Seq and WGS), genome assembly, transcriptome assembly, mitochondrial genome and isolated LC-PUFA sequences can be consulted via NCBI. All accession numbers are indicated in Supplementary Table S1 of Supplementary File 2. Supporting data such as protein and transcripts from genome as well as .gff file of the annotation can be obtained from https://figshare.com/s/98f0644bd974f891143c.
