# Supplementary material for: “Out of the Can”: A Draft Genome Assembly, Liver Transcriptome, and Nutrigenomics of the European Sardine, Sardina pilchardus
Source: Genes (Basel). 2018 Oct 9;9(10):485. doi: 10.3390/genes9100485 (PMC6210256; doi:10.3390/genes9100485)
Supplement: Supplementary file 1 [file genes-09-00485-s001.zip › Supplementary File 1.docx]

**Supplementary File 1**

**“*Out of the can*”: a draft genome assembly, liver transcriptome and nutrigenomics of the European sardine, *Sardina pilchardus***

Authors: André M. Machado ^1,†^, Ole K. Tørresen ^2,†^, Naoki Kabeya ^3,†^, Alvarina Couto ^1,4^, Bent Petersen ^5,6^, Mónica Felício ^7^, Paula F. Campos ^1,4^, Elza Fonseca ^1,8^, Narcisa Bandarra ^7^, Mónica Lopes‑Marques ^1^, Renato Ferraz ^1,9^, Raquel Ruivo ^1^, Miguel M. Fonseca ^1^, Sissel Jentoft ^2,10,^*, Óscar Monroig ^11,^*, Rute da Fonseca ^4,12,^* and L. Filipe C. Castro ^1,8^*

^1^ Interdisciplinary Centre of Marine and Environmental Research (CIIMAR), University of Porto, Matosinhos 4450-208, Portugal, andre.machado@ciimar.up.pt (A.M.); alvarinacouto@gmail.com (A.C.); campos.f.paula@gmail.com (P.C.); fonseca.ess@gmail.com (E.F.); monicaslm@hotmail.com (M.L.-M.); renatobarbosaferraz@gmail.com (R.F.); ruivo.raquel@gmail.com (R.R.); mig.m.fonseca@gmail.com (M.F.)

^2^ Centre for Ecological and Evolutionary Synthesis (CEES), Department of Biosciences, University of Oslo, 0371 Oslo, Norway, o.k.torresen@ibv.uio.no

^3^ Department of Aquatic Bioscience, The University of Tokyo, Tokyo 113-8654, Japan, naokikby@g.ecc.u-tokyo.ac.jp

^4^ The Bioinformatics Centre, Department of Biology, University of Copenhagen, DK-2200 Copenhagen, Denmark

^5^ Natural History Museum of Denmark, University of Copenhagen, DK-1350 Copenhagen, Denmark, [bent.petersen@snm.ku.dk](mailto:bent.petersen@snm.ku.dk)

^6^ Centre of Excellence for Omics-Driven Computational Biodiscovery, Faculty of Applied Sciences, Asian Institute of Medicine, Science and Technology, Kedah 08000, Malaysia

^7^ Portuguese Institute for the Sea and Atmosphere, (IPMA), 1749-077 Lisbon, Portugal, monicafelicio124@hotmail.com (M.F.); narcisa@ipma.pt (N.B.)

^8^ Department of Biology, Faculty of Sciences, University of Porto, 4099-022 Porto, Portugal

^9^ Institute of Biomedical Sciences Abel Salazar (ICBAS), University of Porto, 4099-022, Porto, Portugal

^10^ Centre for Coastal Research, Department of Natural Sciences, University of Agder, 4630 Kristiansand, Norway

^11^ Instituto de Acuicultura Torre de la Sal, Consejo Superior de Investigaciones Científicas (IATS-CSIC), 12595 Ribera de Cabanes, Spain

12 Center for Macroecology, Evolution, and Climate, Natural History Museum of Denmark, University of Copenhagen, Copenhagen, Denmark.

***This Document Includes:***

**Supplementary *Figures***

Supplementary Figure S1 Estimation of genome size, repeat content, and heterozygosity by GenomeScope.

Supplementary Figure S2 Assessment of the genome assembly by comparison of read spectrum and assembly copy number

Supplementary Figure S3 Circular gene map of the mitochondrial genome of *Sardina pilchardus*.

Supplementary Figure S4 Phylogenetic tree of ray-finned fishes estimated from 13 concatenated individual mtDNA protein-coding gene amino acid sequences.

**Supplementary *Material and Methods***

Assembly & assessment of sardine genome

Overall ortholog comparison across four teleost species

Sardine phylogenomics based on mtDNA protein-coding gene amino acid sequences.

Gene orthologs of LC-PUFA desaturation and elongation are present in the sardine genome and transcriptome

**Supplementary *References***

**Description of supplementary files 2,3 and 4**

Supplementary File 2: Additional Tables (XLS)

- Supplementary Table S1. MixS descriptors and accession numbers of tissue samples, raw data and Assemblies of *Sardina pilchardus.*
- Supplementary Table S2. GenomeScope profile statistics for *Sardina pilchardus* WGS reads estimated using k-mer 21, 25, and 31.
- Supplementary Table S3. Completeness assessment of sardine genome using the liver transcriptome.
- Supplementary Table S4. List of species and GenBank references used in the mtDNA phylogeny.
- Supplementary Table S5. Blast-n output of *Clupea harengus* sequences against the sardine genome. These values were used to reconstruct the synteny of the *locus* for each one of the three genes target *fads2*, *elovl2* and *elovl5*.
- Supplementary Table S6. Top results from a reciprocal blast-p search of the 5 proteins flanking the genomic locations of *fads2*, *elovl2* and *elovl5* in herring and the sardine genomes. The 5 sardine proteins were confirmed to be the orthologs of the 5 herring proteins.
- Supplementary Table S7. Primer sequences and PCR conditions used to isolate *fads2*, *elovl2* and *elovl5* genes.
- Supplementary Table S8. Functional characterization of *Sardina pilchardus Elovl2* and *Elovl5* (FA, fatty acid; Nd, not detected)
- Supplementary Table S9. Functional characterization of *Sardina pilchardus* Fads2 (FA, fatty acid; Nd, not detected).
- Supplementary Table S10. *Sardina pichardus* Fads2+Zebrafish Elovl2 co-expression

Supplementary File 3: Clusters of sequences used to Sardine Phylogenomics Analyses (ZIP).

Supplementary File 4: Clusters of sequences used to - Gene orthologs of LC-PUFA desaturation and elongation are present in the sardine genome and transcriptome - analyses (ZIP).

**Additional figures**

**
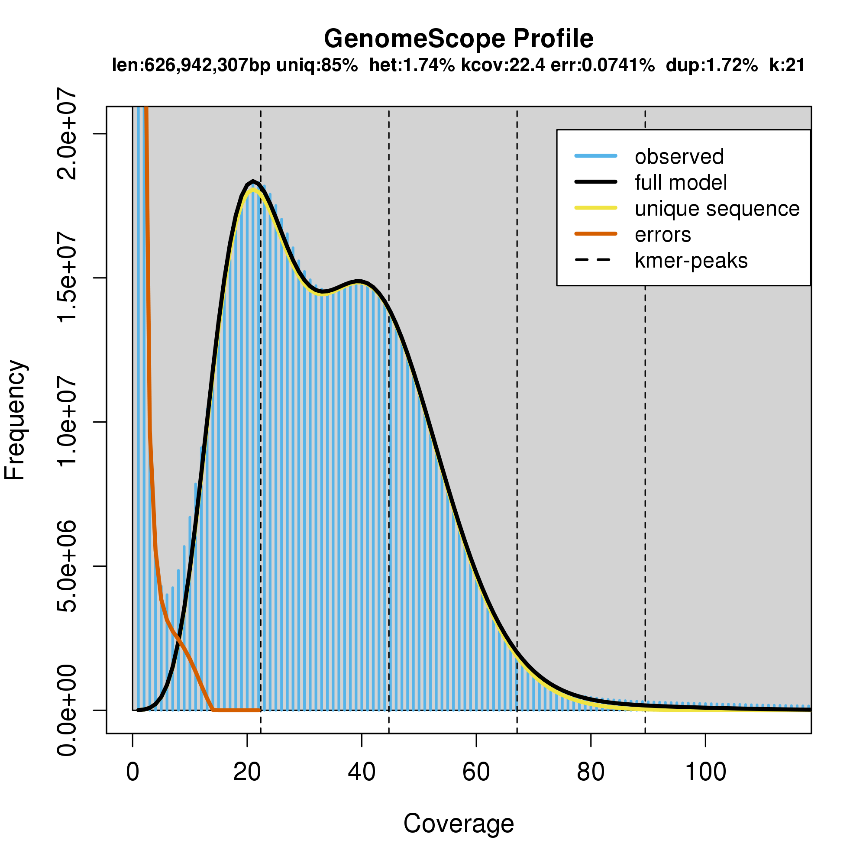
A)**

**B)**

**
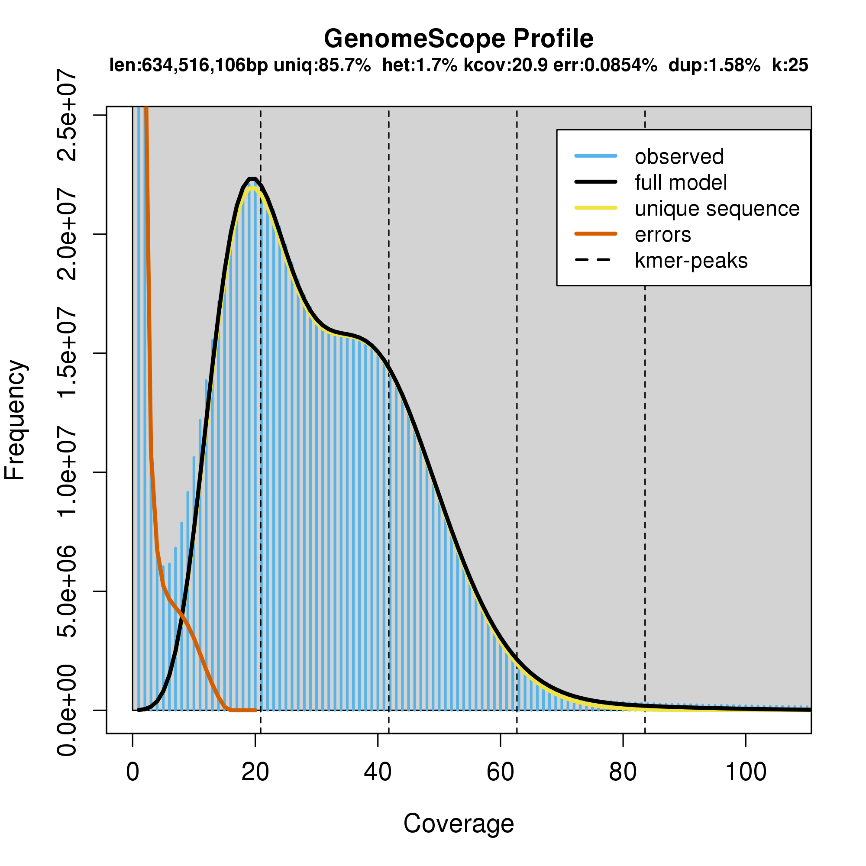
**

**
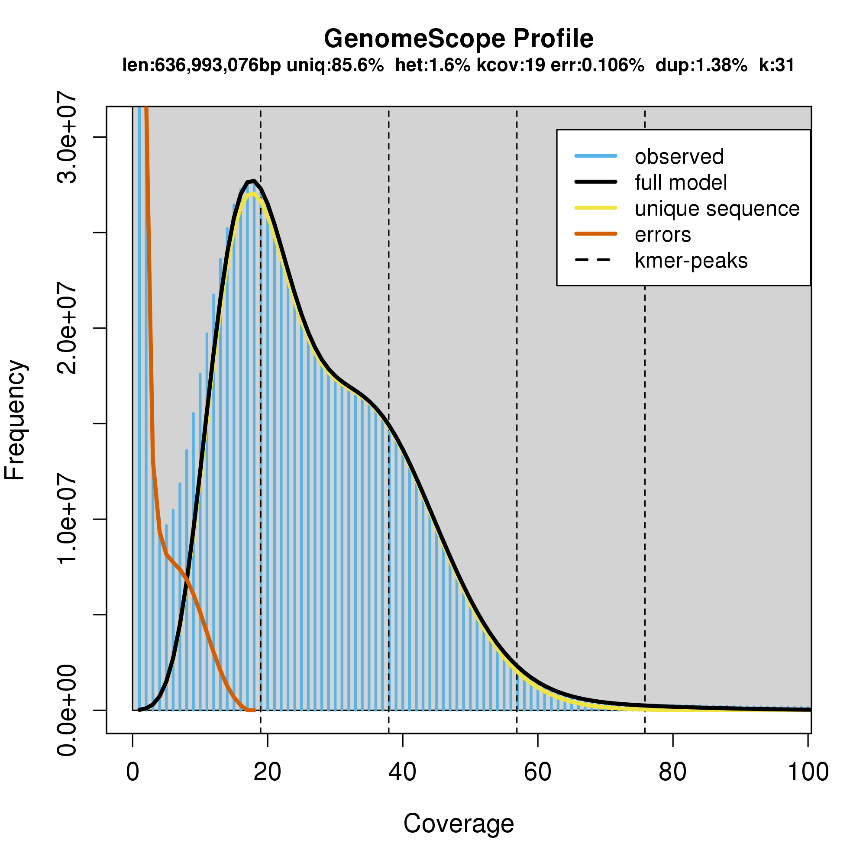
C)**

**Supplementary Figure S1.** Estimation of genome size, repeat content, and heterozygosity by GenomeScope [1], based on 21 (A), 25 (B), 31 (C) k-mers in clean sequence reads (max k-mer coverage at 1000).


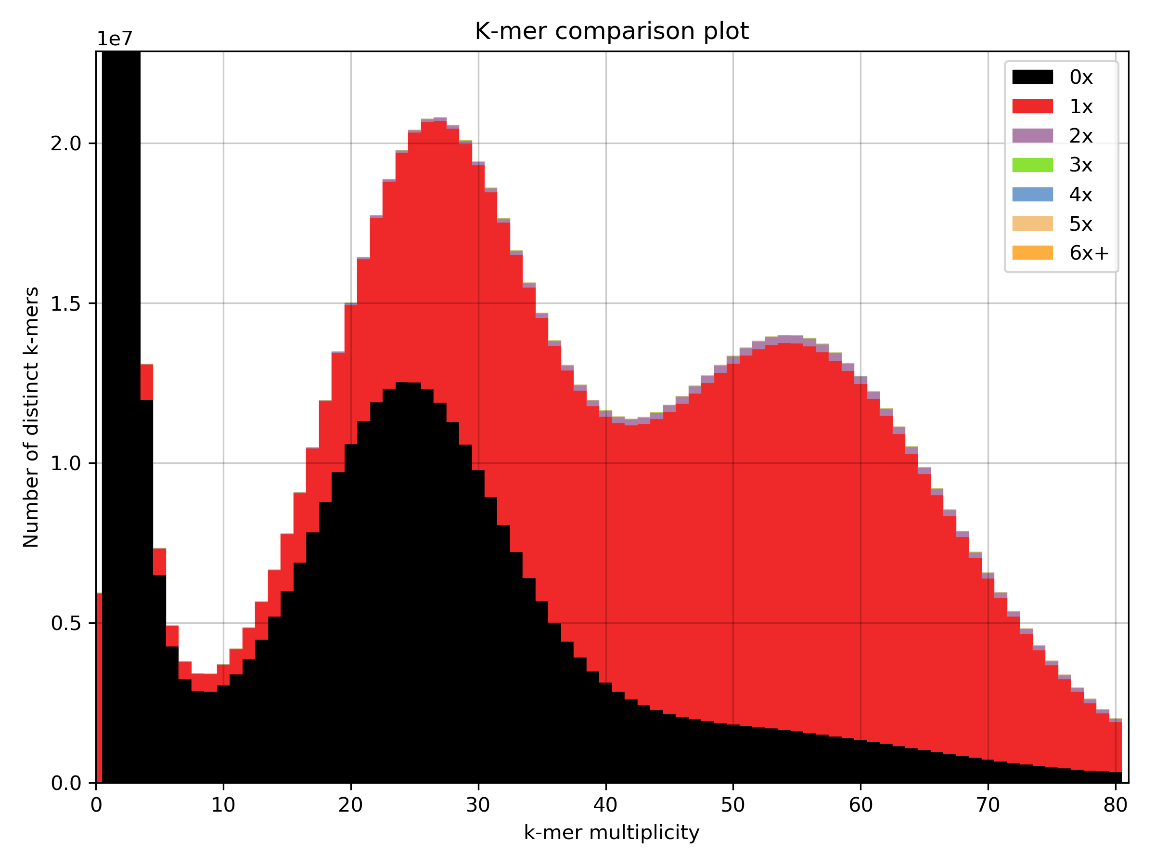


**Supplementary Figure S2.** Assessment of the genome assembly by comparison of read spectrum and assembly copy number. KAT comp tool [2] allowed to observe that the heterozygous content has a representation around of 25x and homozygous content around 55x. Additionally, the major part of shared k-mers , in both peaks, were collapsed during the assembly (black zone), remaining a single copy of the homozygous content and less of the heterozygous content.

**Supplementary**
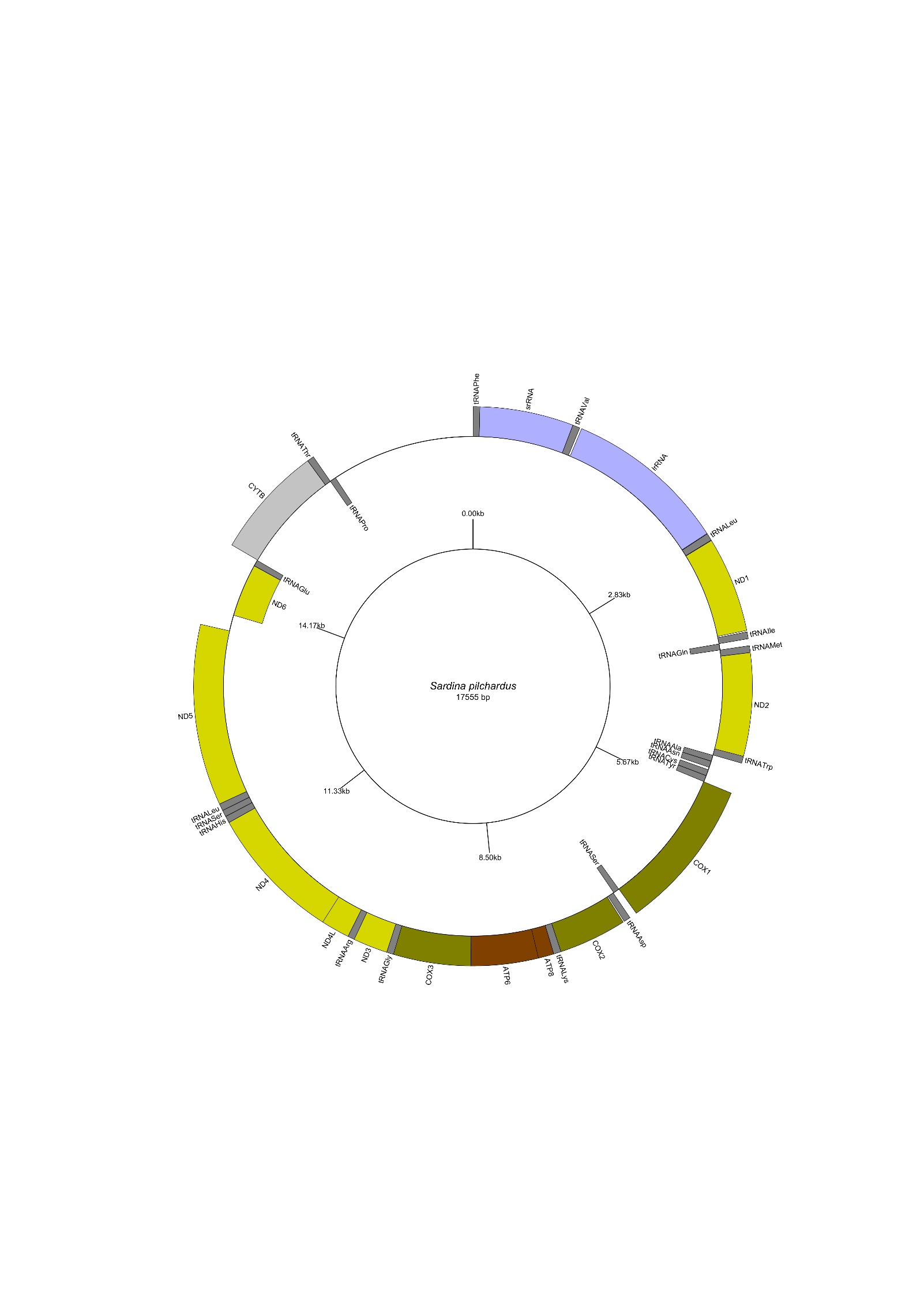
**Figure S3.** Circular gene map of the mitochondrial genome of *Sardina pilchardus*. The circular gene maps were drawn using GenomeVx [3]. Ribosomal genes are coloured in blue, tRNAs in dark grey, and the 13 protein-coding genes are coloured in light and dark green, brown, and light grey.

**Supplementary**
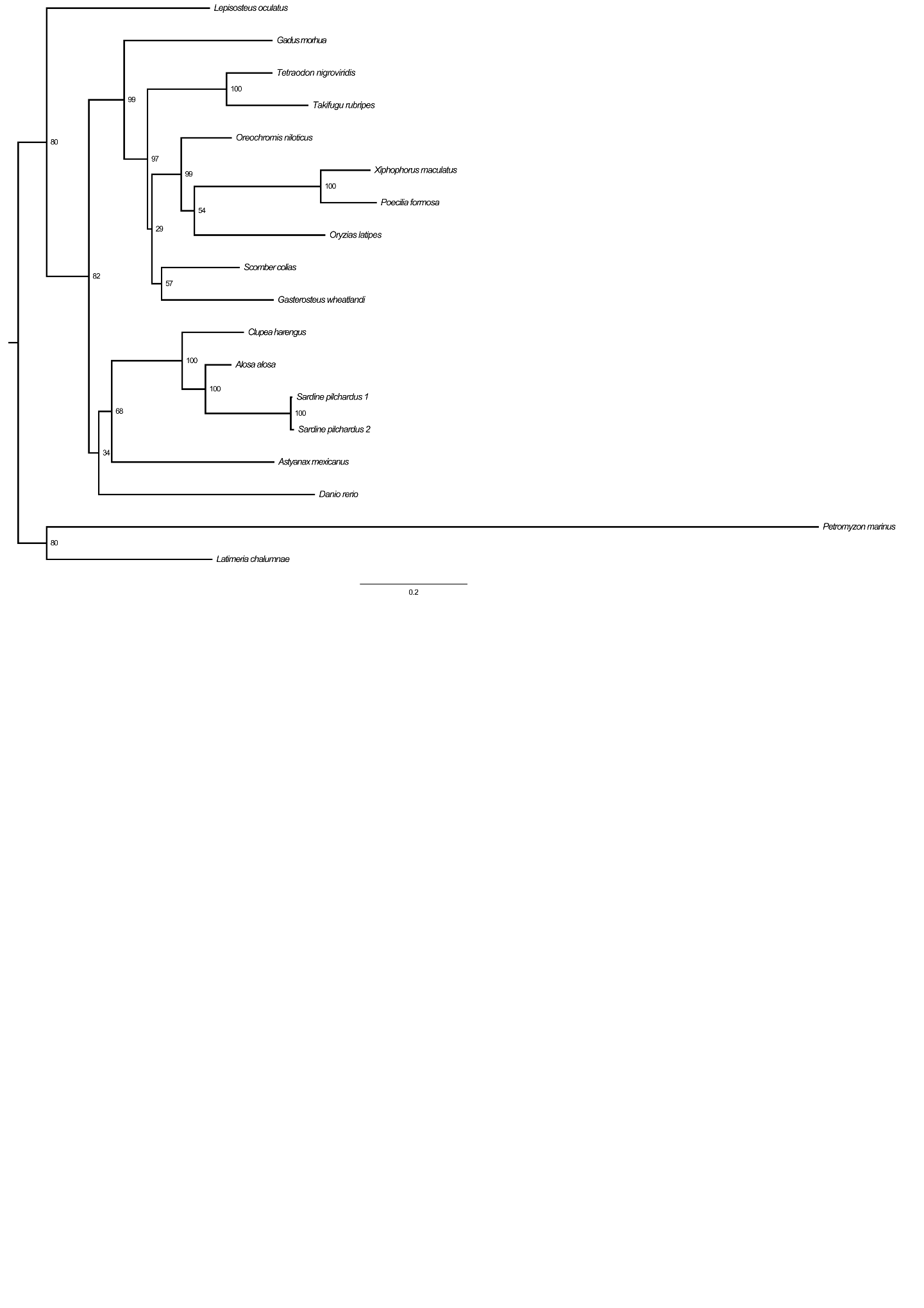
**Figure S4.** Phylogenetic tree of ray-finned fishes estimated from 13 concatenated individual mtDNA protein-coding gene amino acid sequences. Values for branch support correspond to Maximum Likelihood bootstrap support values.

**Supplementary Material Methods**

**Assembly & Assessment of Sardine Genome**

To perform the genome assembly we used the Celera Assembler with the following parameters: (ovlConcurrency = 16 ; ovlThreads =4; cnsConcurrency = 64; merSize = 22; merylMemory = 900000; merylThreads = 64; merThreshold = 0; merDistinct = 0.9995; merTotal = 0.995; doOBT = 0; overlapper = ovl; ovlErrorRate = 0.06; frgMinLen = 64; ovlMinLen = 40; ovlRefBlockSize = 10000000 ; ovlHashBits = 24; ovlHashBlockLength = 800000000; ovlStoreMemory = 500000 ; doFragmentCorrection = 0; unitigger = bogart; utgGraphErrorRate = 0.030; utgGraphErrorLimit= 3.25; utgMergeErrorRate = 0.045; utgMergeErrorLimit = 5.25 ; utgBubblePopping = 1; utgErrorRate = 0.03; utgErrorLimit = 2.5; batThreads = 64; doExtendClearRanges = 0; doToggle = 0; cnsMaxCoverage = 100).

**Overall ortholog comparison across four teleost species**

The annotated gene repertoire were compared with other three teleost fishes using the OrthoFinder v2.2.6 [4] software. To achieve this goal we download the proteome of *Danio rerio* (GCF_000002035.6_GRCz11), *C. harengus* (GCF_000966335.1_ASM96633v1) and *Astyanax mexicanus* (GCF_000372685.2_Astyanax_mexicanus-2.0) from NCBI RefSeq database (ftp://ftp.ncbi.nlm.nih.gov/genomes/refseq/vertebrate_other/). The three proteomes as well as the proteome resulting from the sardine annotation (https://figshare.com/s/98f0644bd974f891143c) were used as input to the OrthoFinder. We used the software with the following parameters: (-t 50 -M msa -S diamond [5]).

**Sardine phylogenomics based on mtDNA protein-coding gene amino acid sequences**

To perform the mitochondrial phylogenetic tree of ray-finned fishes we used the 13-concatenated mtDNA protein-coding sequences of each individual presented in Supplementary File 2, Supplementary Table S4. The gene sequences were aligned with MAFFT v7.309 [6] (model G-INS-i with a maximum number of iterative refinement of 1000) and the resultant multiple sequence alignments (MSA) were trimmed with TrimAl v1.4.rev8 [7] (*automated1* algorithm). Then, the MSA were concatenated and the optimal partitioning scheme was selected (BIC ranking method) using PartitionFinder version 2.1.1 [8] under the greedy algorithm with proportional branch lengths across partitions. Each protein-coding gene was defined as the initial data blocks for the partitioning schemes search. Finally, a Maximum Likelihood phylogenetic inference was performed using RAxML v. 8.0.0 [9] with 100 rapid bootstrap replicates.

**Gene orthologs of LC-PUFA desaturation and elongation are present in the sardine genome and transcriptome**

To address the phylogenetic placement of the identified orthologs we *de novo* assembled the transcriptome of the allis shad (SRA id: SRR1532804) using Trinity [10] with default parameters, and candidate coding regions were identified with TransDecoder [11]. We identified Fads2, Elovl2 and Elovl5 as the top blast-p [12] hits after querying the allis shad’s predicted coding regions against the Atlantic herring’s Fads2 (XP_012687541.1), Elovl2 (XP_012671565.1) and Elovl5 (XP_012695835.1) [13]. To obtain the Fads2, Elovl2 and Elovl5 proteins of the liver transcriptome of sardine we applied the same methodology above explained. The sequences from the longfin inshore squid *Doryteuthis pealeii* (formerly, Loligo) were obtained from the transcriptome available at http://ivory.idyll.org/blog/2014-loligo-transcriptome-data.html. Orthologs for the three genes from three invertebrates (*Helobdella robusta*, *Drosophila melanogaster* and *Octopus bimaculoides*), 12 Actinopterygii and Sarcopterygian species: *Astyanax mexicanus*, *Danio rerio*, *Gadus morhua*, *Gasterosteus aculeatus*, *Latimeria chalumnae*, *Lepisosteus oculatus*, *Oreochromis niloticus*, *Oryzias latipes*, *Petromyzon marinus*, *Takifugu rubripes*, *Tetraodon nigroviridis* and *Xiphophorus maculatu*s, *Homo sapiens* and *Mus musculus*) were obtained from ENSEMBL [14] (Supplementary File 4). The amino acid sequences were aligned with MAFFT [15] with the L-INS-I option. Maximum-likelihood phylogenetic inference was performed with the software RAxML v.8.2.12 [9] for each gene. Tree searches were assessed through 100 bootstrap replicates, under an automatic protein model selection plus gamma, that automatically determines which is the best protein substitution model for each dataset (the one with the highest likelihood score on the parsimony starting tree). To determine the microsynteny of the *fads2*, *elovl2* and *elovl5* in the sardine genome, we used the *C. harengus* genome assembly as reference [16]. Thus, we collected the *C. harengus* CDS of at least one flanking gene of each side of each target gene, and each one it was blasted [12] (blast-n: -word_size 10, -outfmt 6, -num_threads 50) against the sardine genome assembly. After that, we manually inspect the blast-n results and using the qstart, qend, sstart, send and bit score options of outfmt6 format of blast software we reconstruct the loci of each gene (Supplementary File 2, Supplementary Table S5). Additionally, to confirm the neighbors orthology we perform reciprocal blast-p searches of the five proteins, corresponding to five genes flanking the genomic locations of *fads2*, *elovl2* and *elovl5*, in *C. harengus* (*lrrc10b*, *sycp2*, *eps8l2*, *gnal* and *gclc*) (Supplementary File 2, Supplementary Table S6).

To perform the functional characterization of *fads/elovl* in yeast we use the sardine transcriptome assembly. This was searched with blast-n (defaults) using as query *C. harengus* *fads2* (XM_012832087.1), *elovl2* (XM_012816111.1) and *elovl5* (XM_012840381.1) genes. The best scoring transcript for each search was retrieved and aligned with the corresponding gene from *C. harengus*. Sequence alignment inspection revealed that sardine *elovl2* and *elovl5* transcripts were 5´partial transcripts, while sardine *fads2* presented a full ORF transcript. To complete missing regions in *elovl2* and *elovl5* transcripts, a second blast (defaults) search was performed targeting the unassembled transcriptome reads using again *C. harengus* *elovl2* and *elovl5* genes as query. The best scoring reads were collected and upload to Geneious v7.1.9 (https://www.geneious.com), here e*lovl2* and *elovl5* reads were mapped to the *C. harengus* *elovl2* and *elovl5* mRNA sequence, respectively. Reads overlapping with the missing regions were retrieved and assembled to the previously collected sardine transcripts producing a predicated ORF which was later used for primer design. Gene specific primers containing the corresponding restriction sites were designed to isolate each ORF by PCR (Flash High-Fidelity PCR Master Mix, Thermo Fisher Scientific, Waltham, MA, USA) using the *Sardina pilchardus* liver cDNA (Primer sequences and PCR conditions available in Supplementary File 2, Supplementary Table S7). PCR products were analyzed in agarose gel 1% and products with the expected size were excised, purified, digested and cloned into pYES2 (Thermo Fisher Scientific, Waltham, MA, USA) (pYES2-*fads2*, pYES2-*elovl5* and pYES2-*elovl2*). Finally, all constructs were confirmed by Sanger sequencing (GATC Biotech, Constance, Germany).

Functional characterization of *fads*/*elovl* genes was carried out by heterologous expression in yeast *Saccharomyces cerevisiae* as previously described [17]. Briefly, after the transformation of each sardine gene, the resulting transgenic yeast were grown in the presence of one of the following fatty acid substrates: 18:2n–6, 18:3n–3, 20:2n–6, 20:3n–3, 20:3n–6, 20:4n–3, 22:4n–6 and 22:5n–3 for Fads2; and 18:2n–6, 18:3n–3, 18:3n–6, 18:4n–3, 20:4n–6, 20:5n–3, 22:4n–6 and 22:5n–3 for Elovl5 and Elovl2. Additionally, to characterize the desaturase activity of the sardine Fads2 towards 24:5n–3, the transgenic yeast co-expressing the sardine Fads2 and *Danio rerio* Elovl2 were grown in the presence of 22:5n–3, which was elongated by yeast to 24:5n-3 as previously described [18]. Concentrations of the exogenously added substrates were 0.5 mM for C_18_, 0.75 mM for C_20_ and 1 mM for C_22_ as uptake efficiency decreases with increasing chain length [19] . After 48 h culture at 30 ºC, the yeast cells were harvested, washed and total lipid extracted to prepared fatty acid methyl esters (FAMEs) [20] .FAME analyses were performed using Fisons GC- 8160 (Thermo Fisher Scientific, UK) gas chromatograph equipped with a 60 m × 0.32 mm i.d. × 0.25 μm ZB-wax column (Phenomenex, UK) and flame ionization detector. Conversions of exogenously added fatty acid substrates to desaturation or elongation products were calculated as [all product area/(all product area + substrate area)] × 100. Substrate fatty acid conversion for the Δ6 desaturase activity towards 24:5n–3 was calculated using the same formula considering 24:5n–3 as a product of the zebrafish Elovl2 [18].

**References**

1. Vurture, G. W.; Sedlazeck, F. J.; Nattestad, M.; Underwood, C. J.; Fang, H.; Gurtowski, J.; Schatz, M. C. GenomeScope: Fast reference-free genome profiling from short reads. In *Bioinformatics*; **2017**, 33, 2202–2204, doi: 10.1093/bioinformatics/btx153.

2. Mapleson, D.; Accinelli, G. G.; Kettleborough, G.; Wright, J.; Clavijo, B. J. KAT: A K-mer analysis toolkit to quality control NGS datasets and genome assemblies. *Bioinformatics* **2017**, *33*, 574–576, doi:10.1093/bioinformatics/btw663.

3. Conant, G. C.; Wolfe, K. H. GenomeVx: Simple web-based creation of editable circular chromosome maps. *Bioinformatics* **2008**, *24*, 861–862, doi:10.1093/bioinformatics/btm598.

4. Emms, D. M.; Kelly, S. OrthoFinder: solving fundamental biases in whole genome comparisons dramatically improves orthogroup inference accuracy. *Genome Biol.* **2015**, *16*, 157, doi:10.1186/s13059-015-0721-2.

5. Buchfink, B.; Xie, C.; Huson, D. H. Fast and sensitive protein alignment using DIAMOND. *Nat. Methods* **2014**, *12*, 59–60, doi: 10.1038/nmeth.3176.

6. Katoh, K.; Standley, D. M. MAFFT multiple sequence alignment software version 7: Improvements in performance and usability. *Mol. Biol. Evol.* **2013**, *30*, 772–780, doi:10.1093/molbev/mst010.

7. Capella-Gutiérrez, S.; Silla-Martínez, J. M.; Gabaldón, T. trimAl: A tool for automated alignment trimming in large-scale phylogenetic analyses. *Bioinformatics* **2009**, *25*, 1972–1973, doi:10.1093/bioinformatics/btp348.

8. Lanfear, R.; Frandsen, P. B.; Wright, A. M.; Senfeld, T.; Calcott, B. Partitionfinder 2: New methods for selecting partitioned models of evolution for molecular and morphological phylogenetic analyses. *Mol. Biol. Evol.* **2017**, *34*, 772–773, doi:10.1093/molbev/msw260.

9. Stamatakis, A. RAxML version 8: A tool for phylogenetic analysis and post-analysis of large phylogenies. *Bioinformatics* **2014**, *30*, 1312–1313, doi:10.1093/bioinformatics/btu033.

10. Grabherr, M. G.; Haas, B. J.; Yassour, M.; Levin, J. Z.; Thompson, D. A.; Amit, I.; Adiconis, X.; Fan, L.; Raychowdhury, R.; Zeng, Q.; Chen, Z.; Mauceli, E.; Hacohen, N.; Gnirke, A.; Rhind, N.; di Palma, F.; Birren, B. W.; Nusbaum, C.; Lindblad-Toh, K.; Friedman, N.; Regev, A. Full-length transcriptome assembly from RNA-Seq data without a reference genome. *Nat. Biotechnol.* **2011**, *29*, 644–652, doi:10.1038/nbt.1883.

11. Haas, B. J.; Papanicolaou, A.; Yassour, M.; Grabherr, M.; Blood, P. D.; Bowden, J.; Couger, M. B.; Eccles, D.; Li, B.; Lieber, M.; Macmanes, M. D.; Ott, M.; Orvis, J.; Pochet, N.; Strozzi, F.; Weeks, N.; Westerman, R.; William, T.; Dewey, C. N.; Henschel, R.; Leduc, R. D.; Friedman, N.; Regev, A. De novo transcript sequence reconstruction from RNA-seq using the Trinity platform for reference generation and analysis. *Nat. Protoc.* **2013**, *8*, 1494–1512, doi:10.1038/nprot.2013.084.

12. Camacho, C.; Coulouris, G.; Avagyan, V.; Ma, N.; Papadopoulos, J.; Bealer, K.; Madden, T. L. BLAST+: architecture and applications. *BMC Bioinformatics* **2009**, *10*, 421, doi:10.1186/1471-2105-10-421.

13. Lavoué, S.; Miya, M.; Saitoh, K.; Ishiguro, N. B.; Nishida, M. Phylogenetic relationships among anchovies, sardines, herrings and their relatives (Clupeiformes), inferred from whole mitogenome sequences. *Mol. Phylogenet. Evol.* **2007**, *43*, 1096–1105, doi:10.1016/j.ympev.2006.09.018.

14. Kersey, P. J.; Allen, J. E.; Allot, A.; Barba, M.; Boddu, S.; Bolt, B. J.; Carvalho-Silva, D.; Christensen, M.; Davis, P.; Grabmueller, C.; Kumar, N.; Liu, Z.; Maurel, T.; Moore, B.; McDowall, M. D.; Maheswari, U.; Naamati, G.; Newman, V.; Ong, C. K.; Paulini, M.; Pedro, H.; Perry, E.; Russell, M.; Sparrow, H.; Tapanari, E.; Taylor, K.; Vullo, A.; Williams, G.; Zadissia, A.; Olson, A.; Stein, J.; Wei, S.; Tello-Ruiz, M.; Ware, D.; Luciani, A.; Potter, S.; Finn, R. D.; Urban, M.; Hammond-Kosack, K. E.; Bolser, D. M.; De Silva, N.; Howe, K. L.; Langridge, N.; Maslen, G.; Staines, D. M.; Yates, A. Ensembl Genomes 2018: An integrated omics infrastructure for non-vertebrate species. *Nucleic Acids Res.* **2018**, *46*, D802–D808, doi:10.1093/nar/gkx1011.

15. Katoh, K.; Misawa, K.; Kuma, K.; Miyata, T. MAFFT: a novel method for rapid multiple sequence alignment based on fast Fourier transform. *Nucleic Acids Res.* **2002**, *30*, 3059–3066, doi:https://doi.org/10.1093/nar/gkf436.

16. Martinez Barrio, A.; Lamichhaney, S.; Fan, G.; Rafati, N.; Pettersson, M.; Zhang, H.; Dainat, J.; Ekman, D.; Höppner, M.; Jern, P.; Martin, M.; Nystedt, B. B.; Liu, X.; Chen, W.; Liang, X.; Shi, C.; Fu, Y.; Ma, K.; Zhan, X.; Feng, C.; Gustafson, U.; Rubin, C.-J. J.; Sällman Almén, M.; Blass, M.; Casini, M.; Folkvord, A.; Laikre, L.; Ryman, N.; Ming-Yuen Lee, S.; Xu, X.; Andersson, L.; Barrio, A. M.; Lamichhaney, S.; Fan, G.; Rafati, N.; Pettersson, M.; Zhang, H.; Dainat, J.; Ekman, D.; Hï¿½ppner, M.; Jern, P.; Martin, M.; Nystedt, B. B.; Liu, X.; Chen, W.; Liang, X.; Shi, C.; Fu, Y.; Ma, K.; Zhan, X.; Feng, C.; Gustafson, U.; Rubin, C.-J. J.; Almï¿½n, M.; Blass, M.; Casini, M.; Folkvord, A.; Laikre, L.; Ryman, N.; Lee, S. Y.; Xu, X.; Andersson, L. The genetic basis for ecological adaptation of the Atlantic herring revealed by genome sequencing. *Elife* **2016**, *5*, 1–32, doi:10.7554/eLife.12081.

17. Kabeya, N.; Yevzelman, S.; Oboh, A.; Tocher, D. R.; Monroig, O. Essential fatty acid metabolism and requirements of the cleaner fish, ballan wrasse Labrus bergylta: Defining pathways of long-chain polyunsaturated fatty acid biosynthesis. *Aquaculture* **2018**, *488*, 199–206, doi:10.1016/j.aquaculture.2018.01.039.

18. Oboh, A.; Kabeya, N.; Carmona-Antoñanzas, G.; Castro, L. F. C.; Dick, J. R.; Tocher, D. R.; Monroig, O. Two alternative pathways for docosahexaenoic acid (DHA, 22:6n-3) biosynthesis are widespread among teleost fish. *Sci. Rep.* **2017**, *7*, 3889, doi:10.1038/s41598-017-04288-2.

19. Lopes-Marques, M.; Ozório, R.; Amaral, R.; Tocher, D. R.; Monroig, O.; Castro, L. F. C. Molecular and functional characterization of a fads2 orthologue in the Amazonian teleost, Arapaima gigas. *Comp. Biochem. Physiol. Part - B Biochem. Mol. Biol.* **2017**, *203*, 84–91, doi:10.1016/j.cbpb.2016.09.007.

20. Hastings, N.; Agaba, M.; Tocher, D. R.; Leaver, M. J.; Dick, J. R.; Sargent, J. R.; Teale, A. J. A vertebrate fatty acid desaturase with Delta 5 and Delta 6 activities. *Proc. Natl. Acad. Sci.* **2001**, *98*, 14304–14309, doi:10.1073/pnas.251516598.
